# Study of Optoelectronic Features in Polar and Nonpolar Polymorphs of the Oxynitride Tin-Based Semiconductor InSnO_2_N

**DOI:** 10.1021/acs.jpclett.3c00211

**Published:** 2023-02-06

**Authors:** Maurizia Palummo, Michele Re Fiorentin, Koichi Yamashita, Ivano E. Castelli, Giacomo Giorgi

**Affiliations:** † Department of Physics & INFN, Universitá di Roma “Tor Vergata,” Via della Ricerca Scientifica 1, 00133 Roma, Italy; ‡ Department of Applied Science and Technology, Politecnico di Torino, corso Duca degli Abruzzi 24, 10129 Torino, Italy; ¶ Graduate School of Nanobioscience, Yokohama City University, Yokohama 236-0027, Japan; § Department of Energy Conversion and Storage, Technical University of Denmark, DK-2800 Kgs. Lyngby, Denmark; ∥ Department of Civil & Environmental Engineering (DICA), The University of Perugia, Via G. Duranti 93, 06125 Perugia, Italy; ⊥ CIRIAF - Interuniversity Research Centre, University of Perugia, Via G. Duranti 93, 06125 Perugia, Italy; # CNR-SCITEC, 06123 Perugia, Italy

## Abstract

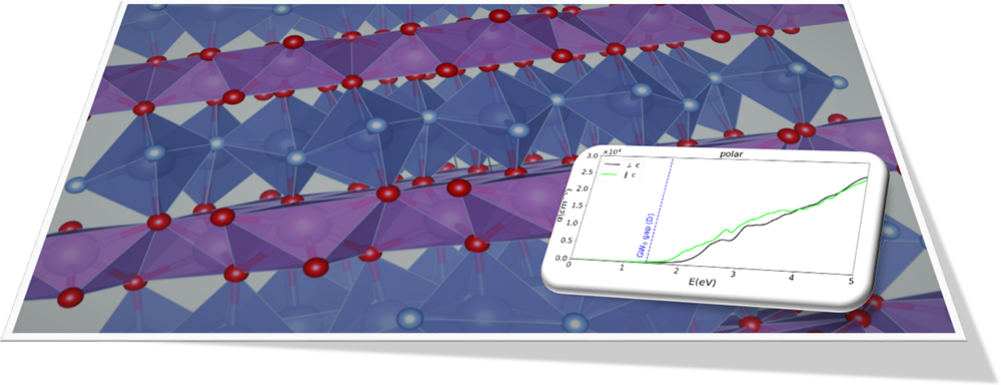

In view of its potential applicability in photoconversion processes, we here discuss the optoelectronic features of the recently proposed tin-based oxynitride material for (photo)catalysis, InSnO_2_N. In detail, by combining Density Functional and Many-Body Perturbation Theory, we compute the electronic and optical properties discussing how they vary from the nonpolar phase to the more stable polar one. After providing a detailed, unbiased, description of the optoelectronic features of the two phases, we have finally calculated the Spectroscopic Limited Maximum Efficiency and obtained data that further witness the relevance of InSnO_2_N for solar energy conversion processes.

The increasing spread of pollutants produced by fossil fuel combustion motivates the scientific community toward the quest for green, environmentally friendly alternatives to such poisoning compounds where the role played by renewable energy sources is relevant. In this respect, the exploitation of solar power has witnessed a breakthrough in the last decades with the appearance of hybrid organic–inorganic halide perovskites (OIHPs),^1^ which have brought the limit of single-junction solar devices very close^2^ to the theoretically predicted limit by Shockley and Queisser,^3^ thanks to the extraordinary features that this class of materials embodies.^4−8^ The mass production of devices exploiting OIHPs still remains challenging, mainly for the presence of a short-chain organic cation in the A-site of the ABX_3_ perovskite structure that enhances the OIHPs instability toward heat and moisture, and, not secondarily, for that of a toxic element, Pb, in the B-site of the most performing representative, CH_3_NH_3_PbI_3_, which represents a serious environmental issue. To overcome such nontrivial shortcomings, several alternatives have been proposed, such as the Sn-based OIHPs^9^ where the oxidation process of Sn (Sn^2+^ → Sn^4+^) is fast and massively enhanced by the presence of the mentioned hydrophilic short-chain organic cation in the A-site with consequent device degradation. The replacement of the organic cation with a metallic one is strategic to allow the thermodynamic stabilization of clean Sn-based perovskites. Keeping central the role of perovskite as the ideal crystal playground structure because of the broad tunability of its chemical composition, scientists have been looking for alternative materials able to provide performances comparable to that of halide perovskites, trying to combine efficiency of operation with respect for the environment. Since the initial works of Marchand et al.,^10,11^ oxynitride perovskites with general stoichiometry ABO_2–*x*_N_1+*x*_ have been the topic of deep investigation both for the basic properties of the materials^12,13^ and for their several potential technological applications, ranging from photocatalysis^14^ to dielectrics,^15^ as well as to pigments.^16^ In parallel, quantum chemistry calculations have been exploited to support experimental findings but also to predict the physical properties of several perovskite oxynitrides,^17−19^ while the ever-increasing availability of computing resources, combined with the development of high-throughput screening procedures, has enabled theoreticians to predict novel yet unexplored and not synthesized materials with properties of interest.^20−24^ In this regard, in view of their applicability in (photo)catalysis,^13,14,25,26^ ABO_2_N perovskites (A = alkaline, alkali-earth, rare-earth, or transition metal cation; B = p-block cation) have been the focus of the analysis of Mishra and collaborators^27^ who, by means of first-principles calculations, have scrutinized the whole field of tin-based oxynitride compounds with perovskite structure. Their results clearly assess the broad applicability of ASnO_2_N (A = Y, Eu, La, In, and Sc) semiconductors for optoelectronic applications because of the combination of some relevant factors, i.e., bandgap in the ideal range for solar light absorption, reduced effective masses, and a marked dynamic stability. This class of materials shows two main polymorphs, a nonpolar phase (*P*6_3_*cm*) and a polar one (*P*3*c*1). The energy differences between these two phases are below 60 meV/atom, so within the metastability limit.^28−30^ The low barrier for switching polarization could be used for improving various properties, such as reducing the overpotentials in the oxygen evolution reaction.^31,32^ Here, in view of the possible applications of InSnO_2_N in green optoelectronic applications, we investigate how many-body effects, such as self-energy bandgap renormalization and electron–hole (e–h) interactions, change the electronic and optical features of such material, analyzing their possible relationship with the material polarity. It is worth stressing that, even if works have been published reporting *GW* calculations about oxynitrides^33,34^ and about oxynitride perovskites,^35^ to the best of our knowledge, this represents the very first analysis focusing on the optoelectronic features of this class of compounds by properly including excitonic and local-field effects.

Ground-state atomic structures have been obtained by means of Density Functional Theory (DFT) as implemented in the VASP code,^36−39^ which is very efficient for structural relaxations using the Blöchl all-electron projector augmented wave (PAW) method.^40^ A generalized gradient approximation (GGA) of Perdew–Burke–Ernzerhof (PBE)^41^ along with a plane-wave cutoff energy threshold of 520 eV is used. A force convergence criterion of 0.02 eV/Å has been selected, while a Gamma centered 6 × 6 × 3 mesh in the Brillouin zone has been employed. In our investigation, we considered both polar (p-InSnO_2_N, *P*6_3_*cm*) and nonpolar (np-InSnO_2_N, *P*3*c*1) geometries of InSnO_2_N, whose optimized structures are reported in Figure 1(a). The insets, in this figure, show the main difference in the two structures that is mainly related to the up-down-down corrugation of the In cations along the *c* axis in the polar structure.^31^ The nonpolar one lacks this indium cations motif, which, in this structure, induces a straightening of one of the ∠Sn–N–SnSn dihedral angles in the *ab* plane (same angle is tilted in the polar structure). As it will be shown in the final part of the manuscript, this induces a different atomic orbital decomposition of the band edge states near the gap. The structural optimization shows a slightly enhanced thermodynamic stability for the polar geometry, which is ∼5 meV/atom, with respect to the nonpolar counterpart, in good agreement with previous works.^31^ Similarly to what was performed in several other recent works published by some of us,^42,43^ we use the code Yambo^44,45^ to perform many-body (MB) simulations. Indeed, it allows us not only to calculate, in an efficient and highly parallelized way, quasi-particle (QP) band structures and absorption spectra at the independent QP (IQP) level of approximation but also to solve the Bethe-Salpeter Equation (BSE), estimating the role of attractive e–h interaction and local-field effects^46−50^ and taking into account the full spinorial nature of the wave functions.^51^ Before carrying out the MB calculations, we have to move to the Quantum Espresso (QE) package,^52^ which is interfaced with Yambo. Starting from the relaxed atomic structures obtained with VASP, we then further relax in a consistent exchange–correlation scheme but using norm-conserving scalar relativistic pseudopotentials^53^ with the full semi-core–shell included in the valence both for In and Sn atoms (with a plane-wave cutoff expansion of 2177 eV). We then carry out both self-consistent and non-self-consistent calculations to obtain Kohn–Sham (KS) DFT eigenvalues and eigenvectors (with a plane-wave cutoff expansion of 1088 eV) needed for MB simulations.

**Figure 1:**
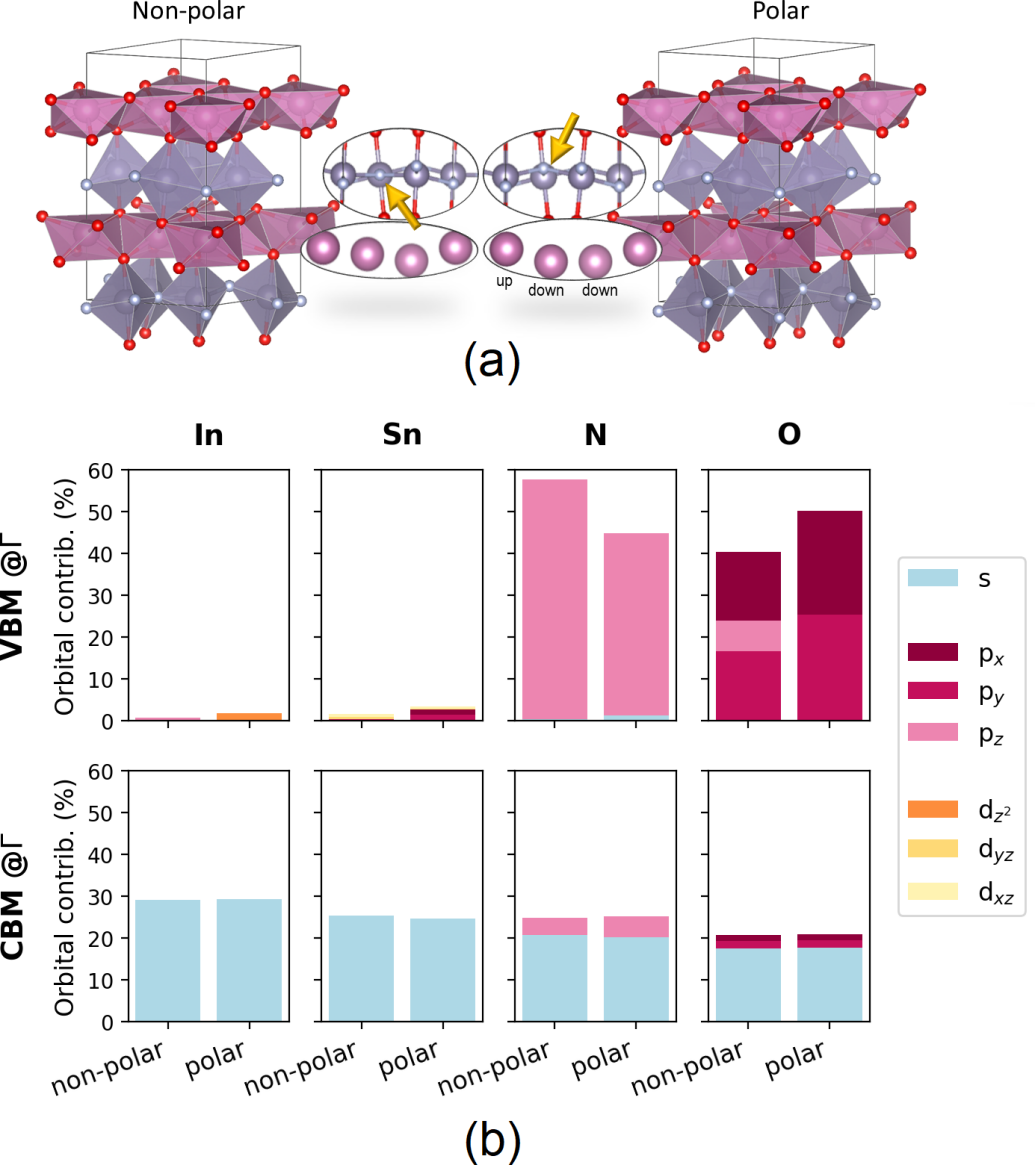
(a) Left, optimized nonpolar and, right, polar structures of InSnO_2_N. For both systems in the inset, the alignment of Sn atoms is reported [large gray, Sn; small silver, N; large purple, In; small red, O atoms]; (b) orbital contribution (%) to the band edges (VBM/CBM) at Γ point for the two, nonpolar and polar, systems.

In detail, for the *GW* analysis a plasmon-pole approximation for the inverse dielectric matrix is applied, with a G-space expansion up to^54^ 109 eV (1088 eV) for the correlation Σ_*c*_ (exchange Σ_*x*_) part of the self-energy (see the Supporting Information section). Several works have shown how the use of a fully self-consistent *GW* scheme,^55−57^ where both the Green function *G* and the screened Coulomb potential *W* are calculated self-consistently, reduces the dependence from the starting point and generally improves the electronic gaps both for molecular systems and solid. Similarly, it has been reported how the use of the partial self-consistent scheme *ev-GW*, updating only the energies both in *G* and *W*, is often an excellent alternative.^56,58,59^ Specifically for the class of perovskites, the QP energies have been obtained either in a fully self-consistent way^60^ or in the *ev-GW* approach, where the self-energy at each iteration, *it*, is evaluated using the energies of the previous iteration, *it-1*: *E*_*n*_^*QP,it*^ = ε_*n*_^*KS*^ + ⟨ψ_*n*_^*KS*^|Σ^*GW*^(*E*_*n*_^*QP,it*–1^) – *v*^*XC*^|ψ_*n*_^*KS*^⟩,^42,61−63^ or, finally, in the *GW*_0_ often on top of hybrid functionals DFT calculations.^64^ Since the dependence of the QP electronic gap, from the starting point, is larger for wide-bandgap materials but would have a minor impact on the excitonic binding energy, we decided to remain at *e-GW*_0_ level of approximation, updating only the energies in *G* while leaving *W*_0_ at the RPA level, using KS eigenvalues and eigenvectors (three iterations are enough to reach convergence, *e-G*_3_*W*_0_; see Figure S3 in the Supporting Information). Further results of *GW*_0_ convergence tests on G-space and sum over unoccupied states, are reported in the Supporting Information (see Figures S3–S5), only for the polar structure. The solution of BSE,^46^ written in transitions (e–h) space, is remapped into the diagonalization of an excitonic Hamiltonian ∑_*e*′,*h*′_*H*_*e,h;e*′,*h*′_^*exc*^*A*_λ_^*e*′,*h*′^ = Ω_λ_*A*_λ_^*e,h*^, where *A*_λ_^*e,h*^, Ω_λ_ are the excitonic eigenvectors, eigenvalues. Within the Tamm–Dancoff approximation,^46,65^ which is known to be well justified for optical spectra of extended periodic materials, *H*_*e,h;e*′,*h*′_^*exc*^ = (*E*_*e*_^*QP*^ – *E*_*h*_^*QP*^)δ_*e,e*′_δ_*h,h*′_ + (*f*_*e*_ – *f*_*h*_)·[*K*_*e,h;e*′*h*′_^*x*^ – *K*_*e,h;e*′*h*′_^*c*^], with *f*_*e,h*_ the state occupation and *K*^*x*^(*K*^*c*^) the exchange (correlation) part of the e–h kernel. In this way, the absorption *Abs*(ω) ∝ ∑_λ_|*D⃗*_λ_|^2^δ(ω – Ω_λ_), where *D⃗*_λ_ = ∑_*e,h*_*A*_λ_^*e,h*^⟨*e*|*d⃗*|*h*⟩ are the excitonic dipole matrix elements. Optically forbidden (allowed) *dark (bright)* excitonic transition λ can then be defined according to the very small (high) intensity of excitonic dipole |*D⃗*_λ_|^2^. Twelve (two) occupied (unoccupied) states are used to build up the excitonic matrix (see Figure S7 for convergence test, reported only for nonpolar case).

At first, we discuss the nature of the electronic properties of the two polymorphs. The band structures initially investigated through the KS-DFT approach (@VASP PBE-D3) are in good agreement with previously published data.^31^ A change from a direct to an indirect nature is confirmed, passing from p-InSnO_2_N to np-InSnO_2_N structure. In detail, the gap for p-InSnO_2_N is at the Γ point (0.481 eV, @VASP PBE-D3), while for np-InSnO_2_N the bandgap is indirect (K → Γ, 0.175 eV) with the direct one at Γ which raises to 0.257 eV (still @VASP PBE-D3). The impact of relativistic effects (SOC) is similarly analyzed, without finding a major impact of such effects on the electronic properties of InSnO_2_N (Figure S1 in the Supporting Information shows the negligible impact of SOC on Sn atoms). We recalculate the electronic band structures using the QE package,^52^ whose results confirm the direct nature of the gap for p-InSnO_2_N (0.48 eV, @QE PBE-D3) and the indirect one for np-InSnO_2_N (0.10 eV), respectively. At the same level of theory, we similarly calculate the effective masses of both electrons and holes. The data reported in Table 1 reveal InSnO_2_N to be preferable as electron transport material, rather than hole conductor, with an effective mass even lower than that of GaAs^66,67^ already at the KS-DFT level of theory. We then perform *GW*_0_ calculations^44,45^ and the obtained band structures are reported in Figure 2, confirming the direct bandgap at Γ for p-InSnO_2_N and the indirect for np-InSnO_2_N: the *GW*_0_ values are 1.42 eV for the former and 0.91 eV for the latter. To complete the scenario and further validate our setup, for the sake of comparison with previous published data, we calculate the bandgap of p-InSnO_2_N by means of the bare HSE06 hybrid functional^68^ finding a value of 1.79 eV, in good agreement with the work of Lan et al.^31^ This reveals a tendency of *GW*_0_ to slightly close the HSE06 electronic bandgap but with the two approaches confirming the excellent properties of p-InSnO_2_N as solar harvester in single-junction devices. Furthermore, the carrier effective masses calculated at the *GW*_0_ level of theory, reported in Table 1, reinforce the message provided by KS-DFT data; i.e., regardless of the polar nature, InSnO_2_N is an excellent electron transport material. As additional comment, we stress that heavy hole masses might be an issue for material exploitation in real devices as the holes might be difficult to extract from the material to the surface where the reactions run. Heavy hole masses is a common issue with both oxides and nitrides semiconductors as inherently n-type doped^27^ due to native anion vacancies, and InSnO_2_N is not an exception in this sense.^21^ Nevertheless, the polar structure might help in photoactivated processes as the intrinsic electric field might facilitate the charges diffusion, potentially delaying the recombination process.

**Table 1:** Calculated Effective Masses for the Two Carriers for Both Polar and Nonpolar Structures^a^

		direction	p-InSnO_2_N	np-InSnO_2_N
DFT	*m*_*h*_	Γ → K	2.81	5.16
		Γ → M	2.81	3.87
		Γ → A	1.44	
		K → H		0.73
	*m*_*e*_	Γ → K	0.113	0.117
		Γ → M	0.113	0.117
*GW*_0_	*m*_*h*_	Γ → K	2.26	4.25
		Γ → M	2.26	3.19
		K → H		0.60
	*m*_*e*_	Γ → K	0.102	0.106
		Γ → M	0.102	0.106

a Results are both at the DFT (@QE PBE-D3) and *GW*_0_ level of theory.

**Figure 2:**
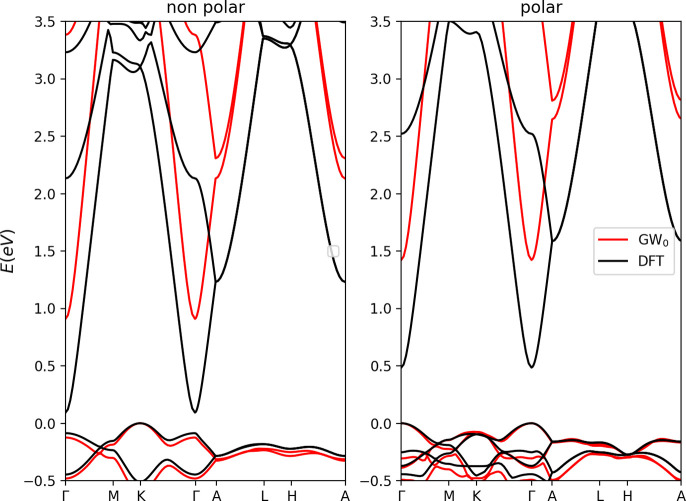
Electronic bandgap calculated for the nonpolar (left) and polar (right) structures of InSnO_2_N. (Γ = 0, 0, 0; K = −0.333, 0.667, 0.0; M = 0.0, 0.5, 0.0; A = 0.0, 0.0, 0.5; L = 0.0, 0.5, 0.5; H = −0.333, 0.667, 0.5) [black, PBE; red, e-*GW*].

We discuss now the optical spectra of the two materials, obtained first at the independent quasi-particle (IQP) (see Figure 3(a)) and then at the BSE level of approximation (see Figure 3(b)). While for the first type of simulations, fully converged spectra in terms of both *k*-points sampling and transitions involved, are reported (see Figure S4–S6 of SI for a complete discussion about convergence), the BSE spectra have been converged only up to 3 eV and are obtained using only a 12 × 12 × 3 *k*-points sampling of the BZ. Figure 3(a) reports, the calculated absorption spectra for light-polarized parallel and perpendicular to the *c* axis. First of all, it is worth noting that the absorption coefficient values are comparable to those of silicon within the IR-VIS region. Moreover, while for np-InSnO_2_N a marked optical peak at about 1.0 eV is present only for light polarized parallel to the *c* axis, the p-InSnO_2_N spectra show a more isotropic behavior and a smoother onset above 1.5 eV. Figure 3(b) shows, only for light polarized parallel to *c* axis, the comparison between IQP and BSE calculated spectra both for np and p-InSnO_2_N but using a 12 × 12 × 3 *k*-grid. The spectra obtained using the Double-Grid technique (DbG)^69^ are also reported for the polar case (dashed lines), showing a small blue shift and smoothing of the main optical features both at IQP and BSE level of approximation. While as expected, switching on the electron–hole interaction induces a red shift and a redistribution of the oscillator strengths, the free-carrier nature of the optical excitations of the two materials is clear considering that all the optical peaks are above the electronic gap values marked with blue lines in the figure. From the analysis of the p-InSnO_2_N BSE eigenvalues and eigenvectors, it results that only the first optical excitation, which is *dark*, has a bound excitonic nature with a binding energy of 0.1 eV (note that the use of denser *k*-points grids and the inclusion of ionic contribution to the dielectric screening should reduce this value), while the first *bright* optical excitation is not a bound exciton being +0.2 eV above the electronic gap. By means of a similar analysis for np-InSnO_2_N, we find that the first *bright* optical excitation is +0.04 eV above the indirect minimum electronic gap. We can then conclude that the optical properties of both phases have a free-carrier nature and confirm their good potentiality in photocatalysis and/or PV where the separation of the photoexcited carriers is an important prerequisite.

**Figure 3:**
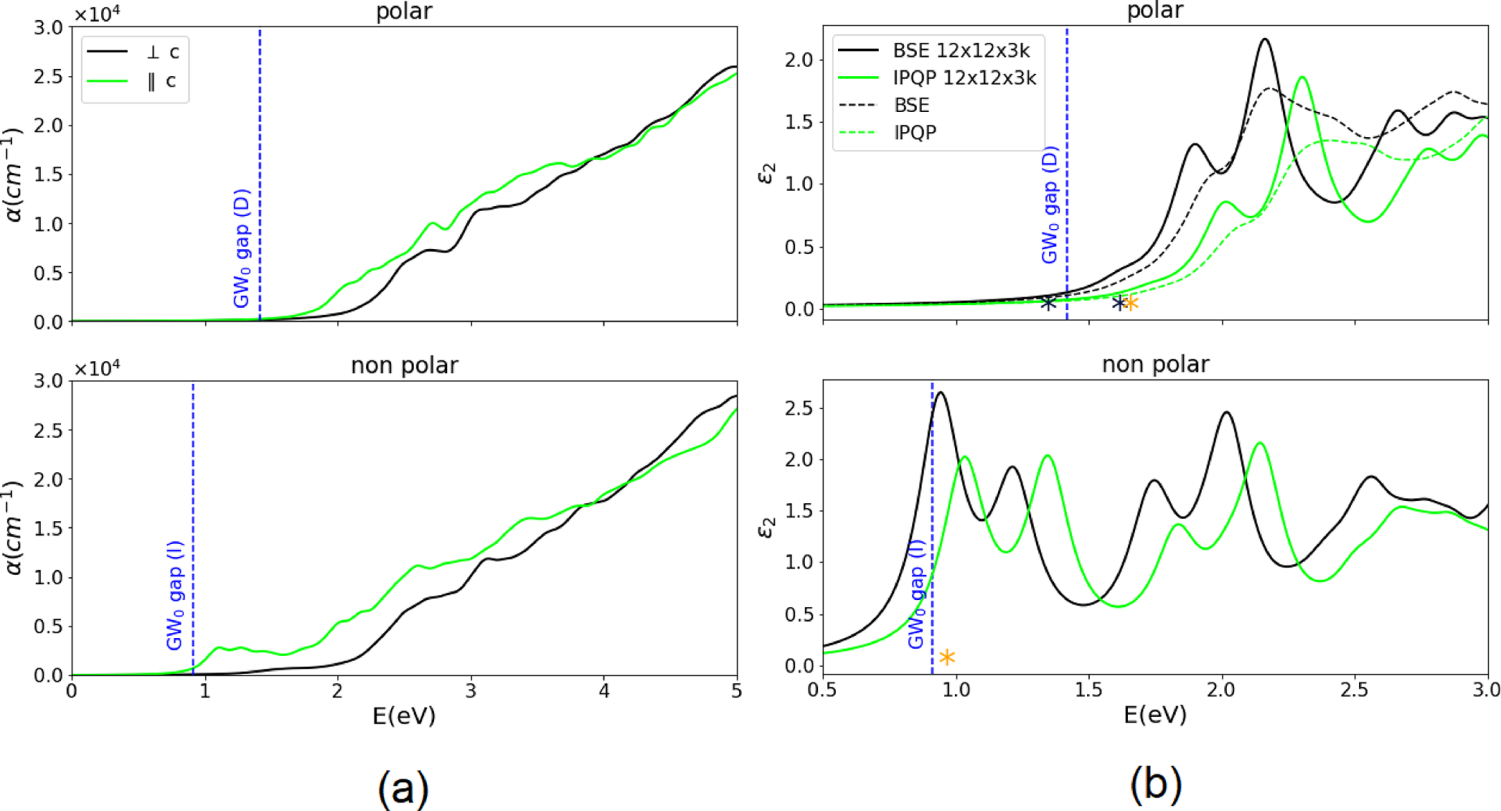
(a) Absorption spectra calculated at the IQP level for polarized light perpendicular (black line) and parallel (green line) to the *c* axis for the polar (upper panel) and nonpolar (lower panel) atomic structures. (b) Optical spectra calculated at the BSE (black) and IQP (green) level for light polarized parallel to the *c* direction for both polar (top panel) and nonpolar (bottom panel) InSnO_2_N structures. Spectra are obtained using 12 occupied and 2 unoccupied states (see Figure S7 of the Supporting Information for convergence tests) in the BSE matrix and a 12 × 12 × 3 *k*-grid. The dashed curves in the top panel are calculated using the method of the Double Grid Technique implemented in Yambo^45,69^ to accelerate the *k*-point convergence. Yellow asterisks: lowest energy bright (direct) excitons. Black asterisks: lowest energy dark (direct) excitons. Dashed vertical blue lines: *GW*_0_ calculated bandgaps (Direct (D), Indirect (I)).

Figure 4 shows the main independent quasi-particle states composing the first dark and bright two-particle optical excitations of p-InSnO_2_N. While the dark ones (indicated with 1,2 in the figure) originate from IQP transitions around Γ from VBM to CBM, the first bright one (3) is due to transitions in the same BZ region but from VBM-1 to CBM. A similar analysis performed for np-InSnO_2_N shows that the first optical excitation, which in this case is bright, is again due to IQP transitions from VBM to CBM. We ascribe the origin of such a polarity-dependent result to the markedly different natures of the band edges of the two systems, and more specifically of the valence band maximum (VBM) near Γ. Indeed, while the conduction band maximum (CBM) at Γ for both np-InSnO_2_N and p-InSnO_2_N is mainly composed of s orbitals of all the atoms forming the system (see Figure 1(b); (polar) In s orbitals 30%, Sn s 24%, N s 20%, O s 17%; (nonpolar) In s orbitals 30%, Sn s 25%, N s 21%, O s 17%), interestingly the VBM of np-InSnO_2_N is maximally localized (53%) on the p_*z*_ orbitals of the two N atoms of the planar, not tilted, ∠Sn–N–SnSn dihedral angle, i.e., that parallel to the *ab* plane (see the np-InSnO_2_N structure inset in Figure 1(a)) with contributions of p_*x*_ (∼16%), p_*y*_ (∼16%), and p_*z*_ (∼16%) orbitals of all oxygen atoms. A negligible amount of d orbitals is observed (Sn d_*xz*_ + d_*yz*_ < 2%; no In d orbitals). On the other hand, the VBM of the p-InSnO_2_N is more delocalized with p_*x*_ (∼25%) and p_*y*_ (∼25%) orbitals of all oxygen atoms equally contributing with an amount comparable to that of all N p_*y*_ orbitals (∼44%). This is another impactful result affecting the optical features of the two phases that we can ascribe to their different, polar vs nonpolar, local geometries. Also in this case the metallic contribution is negligible (In d_*z*^2^_ < 2%; Sn d orbitals formally absent). Figure 1(b) summarizes the orbital contribution of band edges at Γ point, while Figure S2 in the Supporting Information reports the PDOS (in terms of total s, p, and d orbitals) for both polar and nonpolar phases.

**Figure 4:**
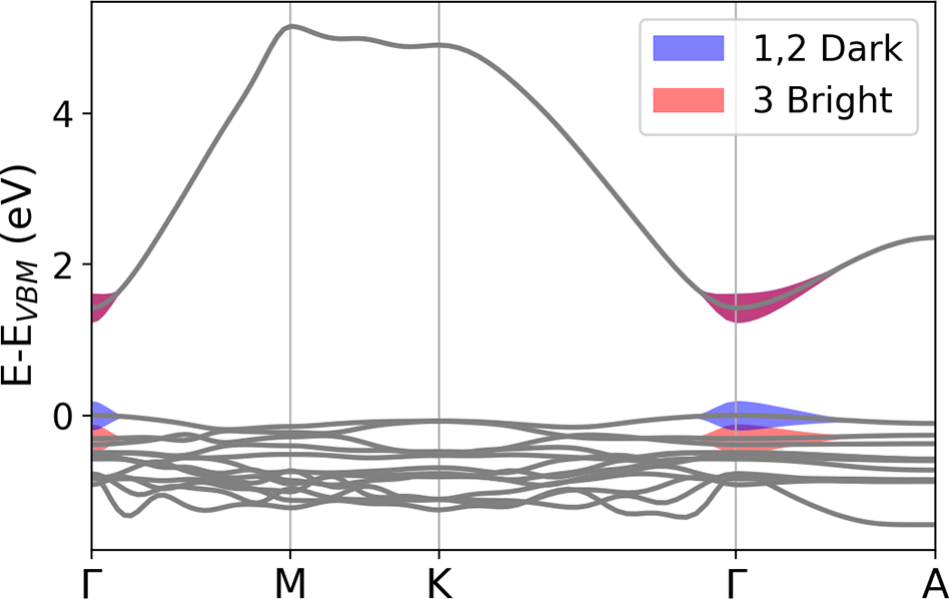
Analysis in terms of independent quasi-particle states of the first optical transitions obtained by solving the BSE for the polar structure.

Regardless of the different (forbidden vs allowed) nature of the first optical transition, such a first transition is characterized by an almost identical spatial (de)localization: we report in Figure 5 the wave function square modulus of the first (optically active, bright) exciton for np-InSnO_2_N. One can notice a quite delocalized electron distribution which tends to be 2D-like with preferential occupation of N atoms, somehow reflecting the population of band edges for the two, polar and centrosymmetric (nonpolar), systems. The large absorption coefficients along with an optimal gap in the visible region assigns to InSnO_2_N a potential, relevant role in photoconversion processes. In this scenario, it could be exploited in photovoltaics and in this regard we calculated the spectroscopic limited maximum efficiency (SLME), a relevant metric associated with the maximum efficiency of the solar harvester in a single-junction solar device.^70^ Additional details of the theory behind the calculation can be found elsewhere.^43,70^ We just remark here that calculating SLME requires several inputs, i.e., the standard solar spectrum, the absorption coefficient of the material, and the values of the direct/indirect electronic bandgaps, while the calculated SLME value is constant for material thicknesses >1 μm. For p-InSnO_2_N, we calculated a value of 30%, while for the np-InSnO_2_N structure the same metric is 27%, both values further witnessing the high potential of the material in photoconversion processes. These values are in line with the best SLME predicted I–III–VI chalcopyrites^70^ and with other, both hybrid organic–inorganic and full inorganic, halide perovskites.^43^ It is worth stressing that, due to the small energy difference (∼0.15 eV) between the two structures, polar (direct bandgap) and nonpolar (indirect bandgap) InSnO_2_N might coexist at room temperature. Interestingly, the calculated SLMEs, which take into account both the absorption curve and the direct/indirect nature of the gap, are very similar for both phases. Clearly, while the polar structure has the advantage that a natural separation of e–h pairs is facilitated by the presence of an intrinsic ferroelectric field, adding cocatalysts could favor the separation of charges in the presence of nonpolar domains.

**Figure 5:**
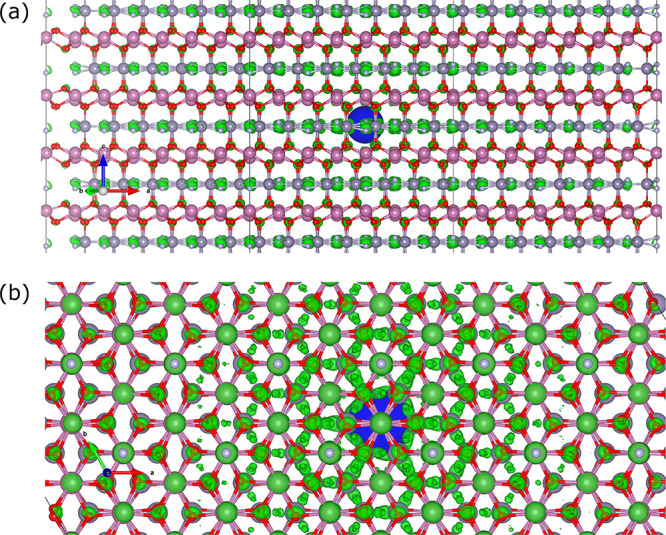
(a) Lateral and (b) top view of the first bright exciton in the nonpolar structure. The green isosurface (1.75 × 10^–7^ e/Å^3^) is the electronic (de)localization associated with the hole position (blue large sphere) [large gray, Sn; small whiteish, N; large purple, In; small red, O atoms].

In conclusion, we have here investigated the optoelectronic properties features of InSnO_2_N, a recently proposed material with promising performances in (photo)catalytic processes. In particular, by combining ground-state Density Functional Theory and state-of-the-art parameter-free excited-state calculations, within the *GW* approach and solving the Bethe-Salpeter equation, we have calculated the absorption spectra for both the polar and nonpolar structure for such a oxynitride compound. Results clearly support the potential usage of such material in photoconversion processes because of an ideal bandgap for single-junction solar devices with characteristics of an electron transport material. The two geometries differ in terms of anisotropy of the absorption spectrum: the nonpolar structure shows indeed a different response as a function of the light polarization, with a transition resonant with the calculated electronic gap, absent in the polar counterpart. We have similarly analyzed the first exciton for the two systems, finding that, for the nonpolar system, the transition associated is permitted (bright exciton), while for the polar one the same transition is forbidden (dark exciton): we ascribe such marked different behavior to the different nature of the valence band, a result clearly dependent on the dual nature, polar vs centrosymmetric, of the InSnO_2_N structure. Our results integrate the previous findings, focused on the catalytic applications of such compounds, by further assessing the great potential of InSnO_2_N in optoelectronics, hopefully opening the way for its synthesis.

## Supporting Information Available

PDOS analysis, the QP energies vs KS ones for p-InSnO_2_N, band number convergence test for the correlation part of self-energy and for screened Coulomb potential, band number convergence test for the bandgap QP energy, absorption spectra at the RPA level for the two structures, plots for the convergence of calculation (PDF)
