# Supplementary material for: Study of Optoelectronic Features in Polar and Nonpolar Polymorphs of the Oxynitride Tin-Based Semiconductor InSnO2N
Source: J Phys Chem Lett. 2023 Feb 6;14(6):1548–55. doi: 10.1021/acs.jpclett.3c00211 (PMC9940202; doi:10.1021/acs.jpclett.3c00211)
Supplement: Supplementary file 1 — jz3c00211_si_001.pdf [file jz3c00211_si_001.pdf]

Supporting Information: Study of Optoelectronic Features in Polar and Non-polar Polymorphs of the Oxynitride Tin-based Semiconductor $InSnO_2N$

Maurizia Palummo*,[†] Michele Re Fiorentin,[‡] Koichi Yamashita,[¶] Ivano Eligio Castelli*,[§] and Giacomo Giorgi*,[∥,⊥,#]

†*Department of Physics & INFN, Universitá di Roma "Tor Vergata," Via della Ricerca Scientifica 1, 00133 Roma, Italy*

‡*Department of Applied Science and Technology, Politecnico di Torino, corso Duca degli Abruzzi 24, 10129 Torino, Italy*

¶*Graduate School of Nanobioscience, Yokohama City University, Yokohama, 236-0027, Japan*

§*Department of Energy Conversion & Storage, Technical University of Denmark, DK-2800 Kgs. Lyngby, Denmark*

∥*Department of Civil & Environmental Engineering (DICA), The University of Perugia, Via G. Duranti 93, 06125 Perugia, Italy*

⊥*CIRIAF - Interuniversity Research Centre, University of Perugia, Via G. Duranti 93, 06125 Perugia, Italy*

#*CNR-SCITEC, 06123 Perugia, Italy*

E-mail: maurizia.palummo@roma2.infn.it(MP);ivca@dtu.dk(IEC);giacomo.giorgi@unipg.it(GG)

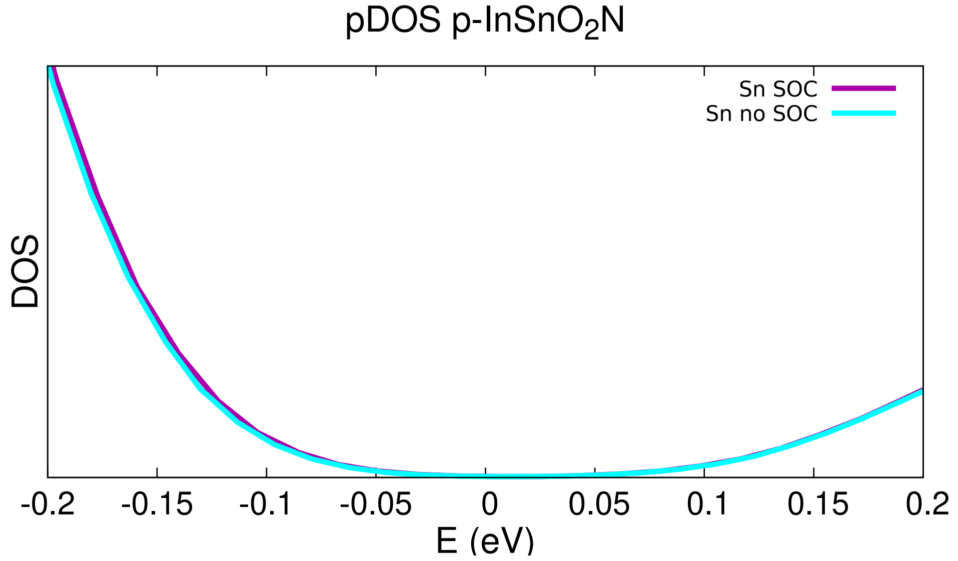

Figure S1: Projected Density of States (PDOS) of the polar structure of $InSnO_2N$ both with (purple) and without (cyan) inclusion of relativistic effects (SOC)

.

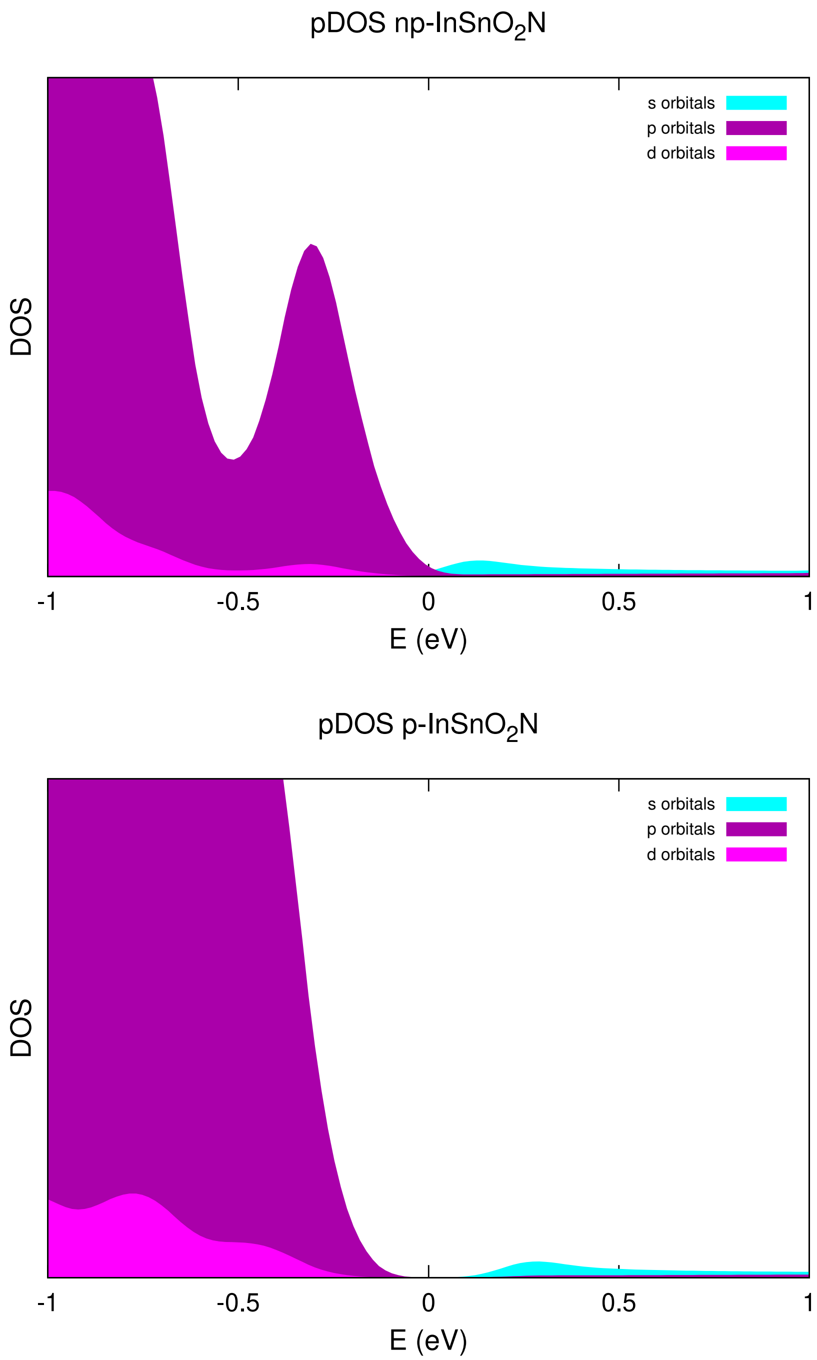

Figure S2: Projected Density of States (PDOS) of the (top) non-polar and of the (bottom) polar phase of $InSnO_2N$

.

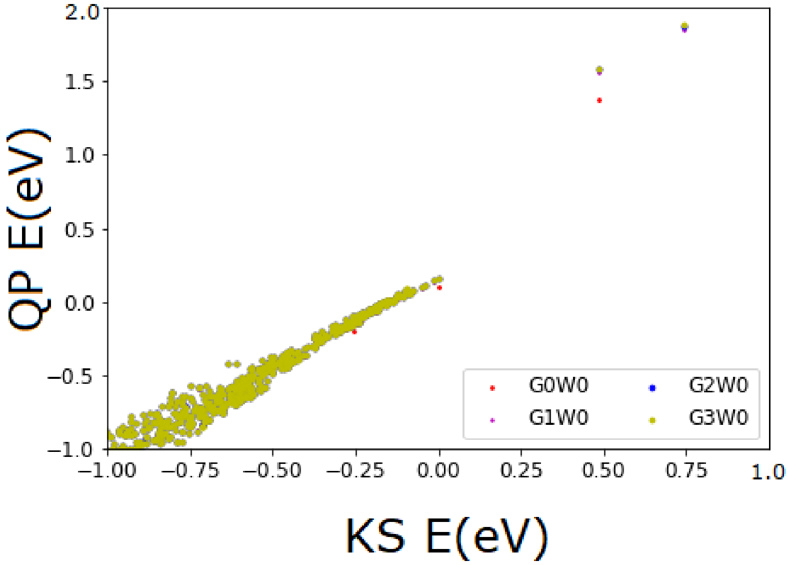

Figure S3: QP energies vs. KS ones for p-$InSnO_2N$. QP energies are calculated at e-G_0W_0, e-G_1W_0, e-G_2W_0, and e-G_3W_0 level of approximation. Similar trend is obtained for np-$InSnO_2N$ and the corresponding plot is not reported here.

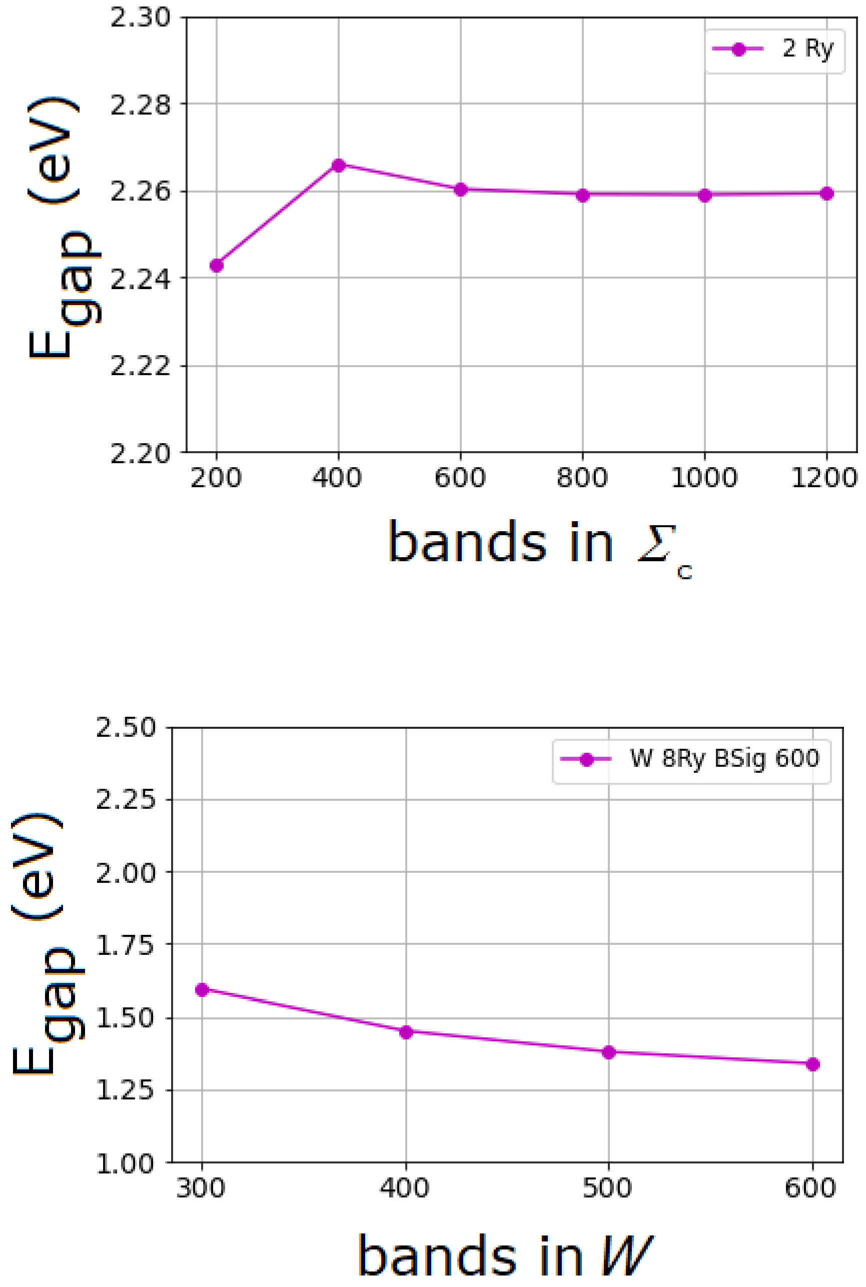

Figure S4: Top, Band number convergence test for the correlation part of self-energy, Σ_c. Bottom, band number convergence test for screened Coulomb potential, W.

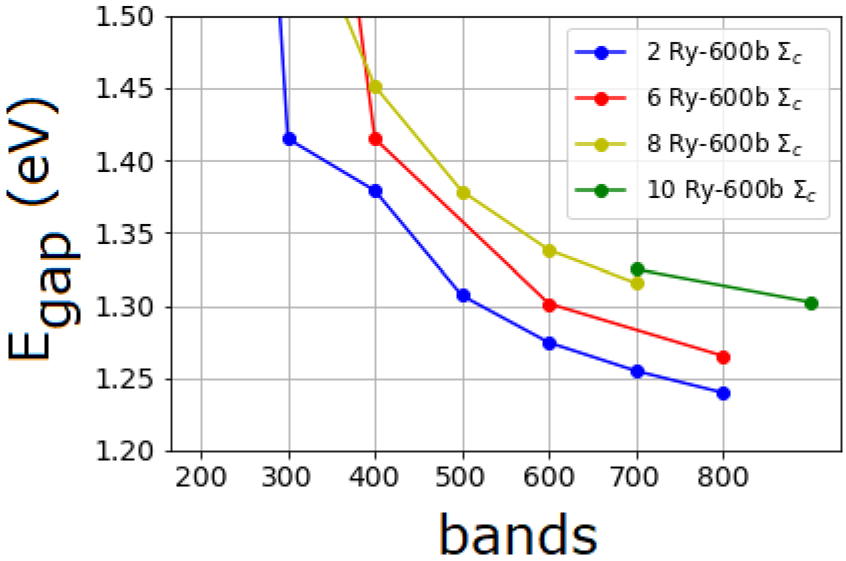

Figure S5: Band number convergence test for the bandgap QP energy

.

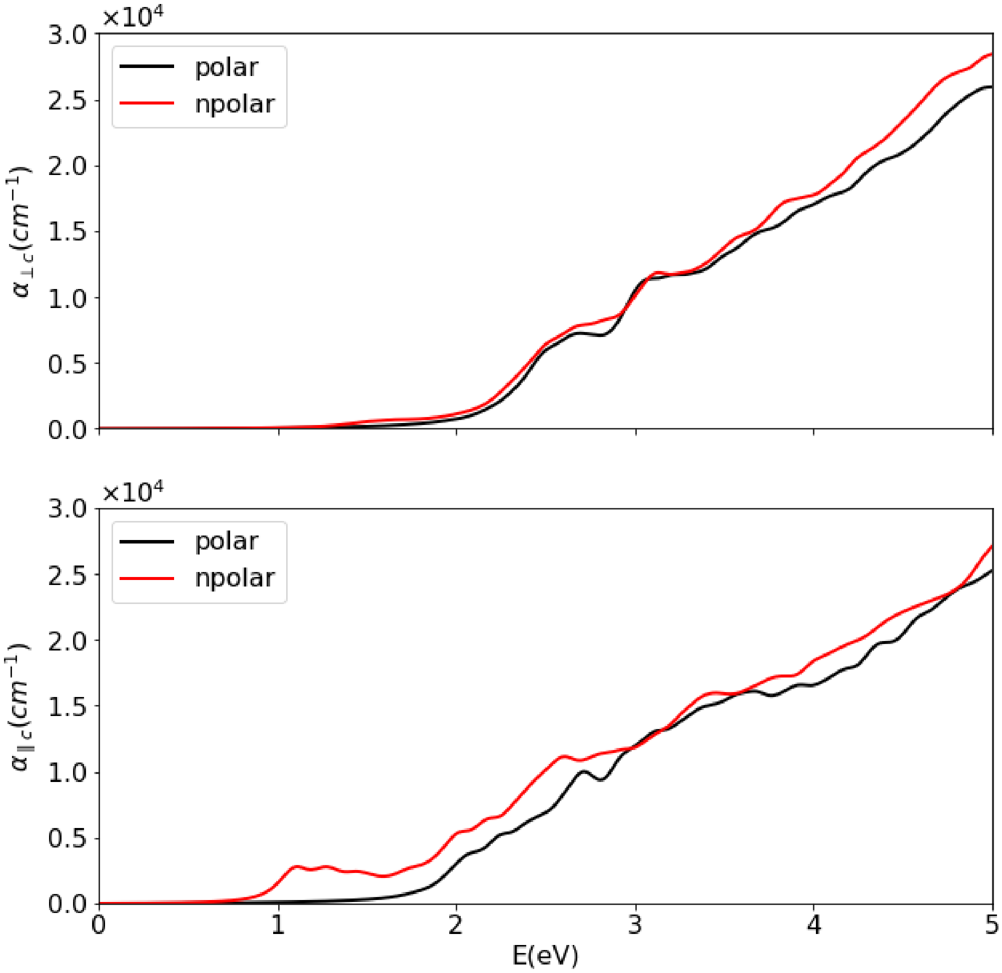

Figure S6: Theoretical calculated spectra at the IP level (RPA) for light polarized perpendicular (a) and parallel (b) to the *c* direction for both polar (black) and non-polar(red) $InSnO_2N$ structure

.

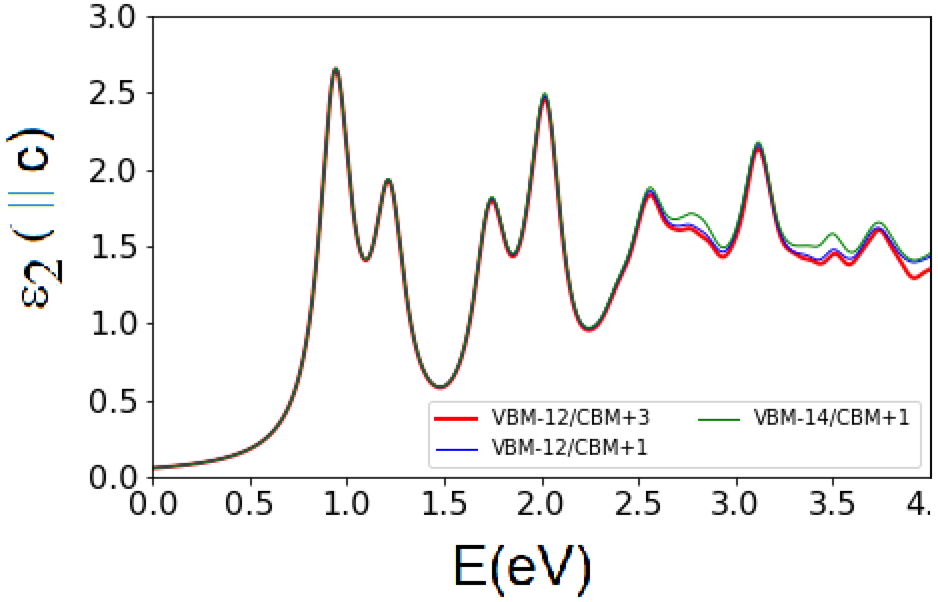

Figure S7: Convergence test of the BSE spectrum in terms of transition (occupied/empty, VB/CB) involved in the excitonic matrix

.
